# General Medicine and Therapeutics

**Published:** 1894-05

**Authors:** 


### GENERAL MEDICINE AND THERAPEUTICS.

EDITED BY DR. LUTHER B. GRANDY.

### WHAT IS THE VALUE OF THE SALICYLATES IN RHEUMATISM?

Many members of the profession will at once answer this question in favor of salicylates. Yet there are others, who are able to quote large statistics and wide clinical experience, who will state that they believe that the general confidence in the value of the salicylates in acute articular rheumatism is not justified by the results which are obtained from its administration.

Probably the correct solution of this mooted point lies in the subdivision of the action of salicylates on the various parts of the body. Under such circumstances there is no doubt that the universal experience has been that salicylic acid or its salts produces a subsidence of the pain and swelling in the joints which are affected by acute articular rheumatism, and that this result may be accomplished after but three or four doses, or certainly by the third or fourth day. On the other hand, the Collective Investigation Committee of the British Medical Association found that the average duration of pain was a little over ten days, while a collection made from the cases treated in the London Hospital barely exceeded five days. Be this as it may, the results of both these investigations, which aggregated something like twelve hundred cases, showed that drugs caused an amelioration of the joint-symptoms. Similarly, salicylic acid produces reduction of the fever, and in many instances the febrile process, under its influences, only persists for two or three days. On the other hand, it must be remembered that every now and then we meet with cases in which the joint-symptoms and the fever resist all doses of the salicylates which we may give within the limits of safety.

So far, then, we may consider that on general principles two of the important symptoms of acute articular rheumatism are relieved.

On the other hand, there are certain complications which are so frequent and occur so early that they may be considered as symptoms or a distinct part of the disease in which salicylic acid probably effects but little,—namely, the appearance of cardiac manifestations of the action of the rheumatic poison. Of course it is true that salicylic acid, by shortening an attack of rheumatism, in that way indirectly protects the heart; but at the same time, there is no evidence that it protects this organ from the actual presence of the disease process. In other words, it may prevent cardiac disease indirectly, but its administration in no way preserves the cardiac apparatus intact while the febrile symptoms last. This has been pointed out in a number of the standard works on medicine, and should be universally recognized by the profession, even though all its members may not be willing to admit that the statement is entirely true. Thus, in Stewart's article in the "System of Therapeutics," he points out that Hood of London, in the treatment of 2,200 cases of acute articular rheumatism appearing in both sexes under thirty-six years of age, noticed that there were a very large number of instances in which cardiac disease was produced. Thus, of 828 cases treated without salicylates, 500 had cardiac disease, whereas in 328 cases treated with salicylates, 190 had cardiac disease; or, again, of 360 cases treated with salicylates, 241 had complications, and in another series 316 suffered from cardiac disease out of a total of 515. In other words, in the cases treated by salicylates the aggregate of cardiac complications was somewhat over sixty per cent., while those who were treated by other measures had cardiac complications in little less than sixty per cent. It is, therefore, evident that the salicylic compounds are no more effective in avoiding these unfortunate manifestations of the rheumatic poison than are the various other modes of treatment. This point is emphasized, too, by the fact that the percentage of cardiac disease in cases treated by the salicylates is about the general percentage of cases,—namely, between fifty and sixty per cent. It is also true that relapses seem to occur more frequently under the salicylates than under the older methods of treatment; perhaps because, when the older methods are used, the disease runs its course, or, as it were, exhausts itself, whereas the salicylates may alter its course before the system is thoroughly rid of the rheumatic poison.

To quote Stewart again: "Garrett has shown that the salicylates seem to have more effect upon the hyperæmic group of symptoms, as, for example, those manifested about a joint, than upon the fibrous symptoms, as, for example, those seen in endocarditis."

There are other points which might be discussed in regard to the value of salicylic acid in rheumatism, but these are, after all, the most important. In view of the recent studies of Haig and those of other investigators who have not been so thorough in their methods of research, we cannot doubt that this class of compounds, uniting with the uric acid, produce salicyluric acid, which is readily eliminated, and so directly or indirectly results in the amelioration of the rheumatic symptoms.

It is hardly in place in this editorial to consider the disadvantages which salicylates possess, so far as their untoward symptoms are concerned. That they are capable of producing disagreeable symptoms every one knows, but they probably do not cause difficulties to arise any more frequently than other drugs possessing medicinal power of great value. There are instances of very severe symptoms, but, after all, these have been few and far between. Perhaps the most noteworthy of these was the case reported within the last few years in the London *Practitioner*, where the use of what might be called a very moderate dose of the salicylates produced petechial eruption and retinal hemorrhages, which later resulted not only in temporary, but permanent blindness.

There are but two points in regard to the use of salicylates in rheumatism which we think should be particularly emphasized, and these are: 1. That the physician, after once deciding to administer these remedies, should give them in large doses or not at all; that is, to use no less than 40 to 80 grains a day. 2. That if, after administering the drugs in this way, no amelioration of the symptoms of any note has occurred at that end of four or five days, that it is useless to continue this line of treatment, as little good will be exercised over the rheumatic process, and much harm will be done by saturating the patient with such irritants to the kidneys and stomach as are the class of the salicylates.—*The Therapeutic Gazette.*

---

### THE MEDICAL CARE OF CHILDREN.

In the treatment of children observation must be our chief reliance. The complexion may be clear and delicately tinted, though pallid, as in the tubercular. If cyanosed, it suggests a respiratory cause; a leaden or earthy tint, especially with great pallor about the upper lip, will frequently be found with gastro-intestinal trouble; the swarthy (*café au lait*), though pallid, face may put us on our guard for syphilis; in the spasmodic stage of whooping-cough the face becomes swollen, often ecchymosed, the eyelids puffy, the conjunctiva conjested and bloodshot; the sunken, vacant eyes, with dark areola around them, indicate great collapse.

The expressin of the face will not infrequently indicate even the seat of disease; so much so that Jadelot has pointed out the following lines in the infant face which, by their position, indicate the seat of derangement: 1. The *oculo-zygomatic line,* from the inner canthus of eye downward and outward to the cheek, a little below the malar eminence, pointing to derangement of the brain and nervous system. 2. The *nasal line,* from the upper part of the ala nasi, curving downward and outward around the corner of the mouth; said to be never absent in gastro-intestinal derangement. 3. The *labial line,* beginning at the angle of the mouth and running downward and outward, generally accompanied by rapidly-moving nares; a fairly trustworthy sign of diseases of the lungs and air-passages.

The child's attitude should also engage attention. A healthy infant lies with limbs semiflexed, and, if on his back, inclines one cheek to rest on the pillow. When the limbs and trunk are rigidly stretched out, tetanus is suggested; when unconscious, or in great weakness and prostration, the child will be found lying flaccid on his back, with face to ceiling. If found on his side, curled up and with head retracted, a frown will probably be seen, together with the oculo-zygomatic line, suggesting cerebral irritation, or, if there is opisthotonos, spinal irritation. If there is intolerance of light, as in cerebral irritation, the face will be buried in the bed-clothes. In abdominal discomfort or in rickets, the child persistently lies on his face, or even rests on elbows and knees.

As regards the cry, some information may also be gained here. Sharp, violent fits of crying, with vigorous movements of the legs, usually indicate colic; the sudden, sharp, single, piercing cry uttered at intervals, while the patient lies in a stupid, drowsy, semi-unconscious state, is but too suggestive of meningitis; while the hoarse, grating cry of syphilis is characteristic. In contrast to these, note when the child does not cry, as in profound weakness brought on by diarrhea, and in grave pulmonary affections where the breath is too precious.

Lastly, we should note both suction and deglutition. Dyspnœa and acute fever cause suction to be performed in short snatches; syphilis also necessitates pauses for breathing. In thrush or ulceration, suction and deglutition are evidently performed with great pain; if the throat be sore, there is frequent cough and noise in deglutition. In great prostration, if the child swallow at all, it is a hopeful sign.—*Dublin Journal of Medical Science.*—*Universal Medical Journal.*

---

### IS TYPHO-MALARIAL FEVER A DISEASE PER SE?

Summarizing, I have reached following conclusions:

First. The fever we have in this part of the country every autumn, and called typho-malarial, is not an acute infectious disease; typhoid fever is.

Second. Typho is probably due to pathological changes, the result of long-continued exposure to malarial influences; or, theoretically, to one or more of the six or eight parasites found in the blood of patients suffering from malarial fevers—to some of these quinine is said to be non-toxic. Typhoid is due to a specific poison.

Third. The incubation and duration of typho are much less fixed than that in typhoid.

Fourth. The temperature range of typho is less uniform and regular than that of typhoid.

Fifth. Diarrhea is often wanting in typho, and rarely absent after the first week in typhoid.

Sixth. The typho tongue never evidences the gravity of disease that the typhoid tongue does.

Seventh. I have never seen sordes on the teeth, gums, lips and in the roof of the mouth, in typho; I have, frequently, in typhoid.

Eighth. I have never discovered the typhoid eruptions on persons of typho-malarial patients. It occurs in eighty-eight per cent. of typhoid patients.

Ninth. Bronchial and pulmonary symptoms are rare in typho; common in typhoid.

Tenth. The nervous symptoms in typho are absolutely wanting, in comparison to their constancy and gravity in typhoid.

Eleventh. The gut pathology of typhoid fever, as discoverable by the symptoms, exists in a much smaller percentage of cases in typho than in typhoid.

Twelfth. By the intestinal dejecta, typhoid fever is disseminated; there is no evidence that this is true of the dejecta of typho.

Finally. Even if the so-called typho-malarial fever of this country is really typhoid fever, the abandonment of the name typho would in the present status of our knowledge, be premature.—*F. L. Sim, M.D., in Memphis Medical Monthly, February*, 1894.

---

### CALOMEL IN HEPATIC ASCITES.

Palma (*Therap. Monatshefte*) points out that Jendrassik in 1885 was the first to recall attention to the diuretic properties of calomel in hydropic conditions, and it has been employed in cardiac, nephritic and hepatic dropsies, giving, in the first condition, valuable results at the hands of most observers. Nephritic complications, when thus treated, did not yield so satisfactorily, whereas opinions differed with regard to the benefits to be derived from calomol in hepatic affections. The author describes a series of eight cases of liver disease, comprising patients with and without ascites, in which this condition, when present, was secondary. Most valuable histories and tables are reproduced, showing a remarkably beneficial result in four of the six ascitic cases, the urine being increased three to tenfold in quantity, causing disappearance of the fluid in the abdominal cavity and all subjective symptoms. In the two remaining subjects with ascites treatment showed no results, the patients dying while in hospital of advanced disease and cholæmia. Very slight diuresis only, though attended with improvement, was produced in two patients, whose disease was unaccompanied by any evidence of œdema or ascites, but great relief was afforded with increase of urine to a ninth and last patient suffering from ascites due to secondary carcinomatous disease of the liver. The calomel was given during successive periods of three days, separated by intervals of one to three days, the drug being given either in repeated doses of four and one-half to nine grains daily, or in quantities decreasing from fifteen to six grains per diem. Two periods sufficed in the cases quoted, and stomatitis and diarrhea were obviated by means of chlorate of potash gargles and opium, the latter only failing rarely and temporarily. Moreover, no renal or cardiac irritation was produced. The manner of action is not quite evident to the author, who, however, inclines to the view that calomel has a direct action on the kidneys and liver, as the failure of the drug in two of his cases to produce diuresis must be attributed to the advanced disease of the liver, no kidney lesion being found *post mortem*.—*British Medical Journal Epitome.*

